# Mesoporous silica chip: enabled peptide profiling as an effective platform for controlling bio-sample quality and optimizing handling procedure

**DOI:** 10.1186/s12014-016-9134-9

**Published:** 2016-11-22

**Authors:** Kai Liang, Hongmei Wu, Tony Y. Hu, Yan Li

**Affiliations:** 1 Laboratory of Interdisciplinary Research, Institute of Biophysics, Chinese Academy of Sciences, Beijing, 100101 China; 2 GuangDong Bio-healtech Advanced Co., Ltd, Foshan City, 52800 GuangDong Province China; 3 Department of Nanomedicine, The Methodist Hospital Research Institute, Houston, TX 77030 USA; 4 Department of Cell and Developmental Biology, Weill Cornell Medical College of Cornell University, 445 E. 69th Street, New York, NY 10021 USA

**Keywords:** Silica mesoporous material, MALDI MS, LMW peptide, Sample quality, Pre-analysis handling

## Abstract

**Background:**

High quality clinical samples are critical for meaningful interpretation of data obtained in both basic and translational medicine. More specifically, optimized pre-analysis handling to bio-sample is crucial for avoiding biased analysis in a clinical setting. A universally applicable method for the evaluation of sample quality and pre-analysis handling is therefore in great demand.

**Methods:**

The fingerprint pattern of low molecular weight (LMW) peptides in sera is directly associated with sample quality and handling process. Previous studies for enrichment/isolation of LMW peptides have shown that LMW peptides can be enriched by silica meso-porous material in a sensitive and high-throughput manner. Here, a peptide profile approach utilizing mesoporous silica chip-based sample preparation combined with MALDI MS analysis was used as a new platform for evaluation of bio-sample quality. Rat sera were selected as model sample and analyzed according to their LMW peptide fingerprint spectra.

**Results:**

This novel method can complete the entire sample preparation procedure in a short period of time (<40 min), requires minimum amounts of sample (<10 µL), is of high sensitivity (LOD 10 ng/mL) as well as high reproducibility (CV% < 15%). According to the acquired LMW peptide spectra, we were able to distinguish the serum samples processed under different conditions (including different storage temperature, time, and freezing/thaw cycles) with the help of bioinformatics tools (principle composition analysis and significant difference analysis), and identify the samples that had significantly changed due to the inappropriate processing. Based on the percentage of significantly changed peaks in LMW peptide mass spectrum after handling, a judgment standard was established that can be used to evaluate the status of preservation of a biological sample. In addition, our principle study established recommendations for storage time, storage temperature and freeze/thaw conditions.

**Conclusion:**

Our novel method for analysis of bio-samples allows for effective identification of variations in composition within samples, and provides a cost-effective tool for simple sample manipulation in a clinical setting.

## Background

Both basic and translational medical researches depend on the availability of high quality clinical samples, which often have to be processed and analyzed in a high-throughput manner [1]. A major issue for biomarker studies, serum samples often become unstable during the collection and storage process, resulting in poor reproducibility. Because of this, few biomarkers proposed have actually been translated into the clinical practice [2–4]. Serum is a highly dynamic biological sample, and contains various degradative enzymes such as proteases. They can be released from activated, dying, or lysed neutrophils or mononuclear phagocytes during sample process [5]. Inevitably, these enzymes will hydrolyze serum proteins and peptides, and as a consequence change the proteomic pattern of the sample. In addition, other processes such as protein/peptide precipitation, aggregation or adsorption can also greatly alter the proteome composition of serum [1]. These alterations are likely influenced by handling conditions, including clotting procedure, storage time, storage temperature and freeze/thaw cycles and other factors [2]. Thus, evaluation of the changes of serum composition and optimization of the sample handling procedure for minimizing the pre-analysis variability are of paramount importance for clinical research.

Ideally, every sample collected for clinical research should be tested or monitored during the entire process of collection, storage, and experimental handling to ensure the highest possible sample quality. Considering the large number of specimens commonly involved in clinical tests, the testing methods used should ideally be fast, consume little sample volume, compatible with high-throughput technology, available for easy manipulation, and cost-effective; besides, the results of the sample analysis should reflect the changes that have occurred in the sample since collection.

Various methods have been developed and applied to assess the quality of clinical sera and to evaluate sampling procedures, including enzyme-linked immunosorbent assay (ELISA) [6, 7], capillary electrophoresis (CE) [8], 2-D or 1-D polyacrylamide gel-electrophoresis (PAGE) [1, 9, 10], antibody microarray chip [10], aptamer microarray array [11], and novel mass spectrometry technologies such as the surface-enhanced (SELDI-) [12–14] and matrix-assisted laser desorption/ionization time of-flight (MALDI-TOF) MS [2, 3, 15–19]. However, there remains a lack of quality-controlling platforms for large-scale analysis of clinical specimen. For instance, ELISA [6, 7] and CE [8] only monitor alterations of one or few proteins, and they do not necessarily reflect variations in sample composition in a comprehensive manner. Furthermore, PAGE profiling has been to be resistant and insensitive to certain changes due to long-term storage and freeze/thaw cycling [1, 10, 20]. The extensive application of microarray technology for quality control in clinical bio-banks is limited by antibodies commercially available, as well as by the high costs associated. SELDI- and MALDI-TOF MS offer great potential for proteome profiling, and have become promising tools for the control of serum quality and assessment of sample handling artifacts. For MS-based protein profiling of clinical samples, commonly used preparation methods are C4/C18 zip-tip resin [2, 15], C8/C18-derivatized magnetic beads [3, 16–19, 21], or anion-exchange chromatography purification [12]. However, these methods are labor- and time-intensive due to the labor-intensive procedures involved, including repeated centrifugation steps, multiple steps of sample transfer, washing and elution, and are therefore of limited use in the clinical settings, where sample numbers usually reach several hundreds or even more. More importantly, highly abundant proteins with large molecular weight (>10 kD, i.e. albumins and globulins) are often over-represented in samples, which in methods without appropriate selection procedures results in under-representation of low-weight peptides or proteins. Thus, a universal method is still required to ensure high sample quality and optimal pre-analysis handling, for instance, appropriate sample preparation and applicable to extensive clinical practices.

Selection of an appropriate target is crucial for meaningful monitoring of serum sample quality, as it is difficult to test every compound in complex bio-fluids. Low molecular weight (LMW) peptides are important resource for disease biomarkers [22–26]. Meanwhile, LMW fraction varies during the sample handling due to the appearance of new peptides that are generated from the hydrolysis of large proteins [18]. Therefore, LMW peptide profiles should be informative about changes in sample composition. Here, we aim to develop a novel approach that is based on the LMW peptide profile for the evaluation of sample quality and handling procedure.

Nano-materials with novel physicochemical properties have been widely used in the area of biomedical research. Mesoporous silica materials (MPSM) are known to take up peptide molecules below certain molecular weights with great efficiency, thanks to their ability to sieve molecules of different sizes. These captured peptides can then be eluted conveniently from the mesoporous silica [22–28]. In addition, it has also been shown that peptides stored in the pores of MPSM exhibit greater stability during long-term storage [28]. Therefore, methods based on MPSF should provide an ideal platform for the enrichment of LMW peptides in clinical samples. Subsequently, these samples can then be analyzed by mass spectrometry to profile the LMW peptides, producing a fingerprint spectrum, which should offer crucial information about the sample composition and its variability.

In this report, we describe the mesoporous-material based MALDI MS approach as a novel tool to assess the quality of clinical specimens, and also to evaluate the handling procedure. We performed a systematic analysis of rat serum to investigate the influence of several pre-analytical factors (including storage temperature, storage time and freeze/thaw cycles) on serum LMW peptide. We analyzed hundreds of samples using a high-throughput procedure in a very short time with little sample consumption, high sensitivity and good reproducibility. Employing bioinformatics tools such as principle composition analysis (PCA) and significant difference analysis, the alterations of samples handled under different conditions can be effectively recognized, and the extent of change of LMW peptide pattern is highly related to handling condition, proving the effectivity of this approach as an evaluation tool. Based on this study, we offer some simple recommendations to avoid or correct systematic bias generated from the sample handling procedure.

## Experiments and materials

### Chemicals

Trifluoroacetic acid (TFA), acetonitrile (CAN, HPLC grade), α-cyano-4-hydroxycinnamic acid (CHCA), synthesized standard peptides (Mw 1338) and the Grace Bio-Labs reusable CultureWell (TM) gaskets were purchased from Sigma-Aldrich (St. Louis, Mo, USA).

### Sample preparation with mesoporous silicon wafer

The mesoporous silicon wafer was prepared according to previously published methods [22–28]. The type of the mesoporous silicon wafer we used was L121 [28], which is a silica material that exhibits large porosity (~55%), with an average pore size of 5.2 nm. The L121 mesoporous silicon wafer could completely exclude proteins over 29,000 Da such as albumin, and help to significantly increase MS sensitivity of peptides below 3500 Da [28].

Serum samples were prepared according to a previously published procedure [23]. The fresh chips were firstly pre-baked overnight in an oven at 120 °C. Afterwards, cultureWell(TM) gaskets were cleaned with 100% ethanol and pressed on the chip surface with forceps to ensure a complete seal with chip. Then 6 μL of serum sample was pipetted into each 3 mm well, and incubated for 20 min in a humidified chamber at room temperature. Then the serum was pipetted up from wells and discard. To wash away the large proteins, 10 μL of deionized water (DI water) was pipetted into each well and then discard directly. The washing step was repeated 4 times. Finally, 6 μL elution buffer (0.1% TFA + 50% ACN) was pipetted to each sample well, incubated in a humidified chamber at room temperature for 90 s, and pipetted out ready to perform MALDI-TOF analysis. The acidic elution buffer could ionize the captured peptides in silicon pores and then efficiently resolved these peptides from pores. Meanwhile, the constitution of elution buffer (0.1% TFA + 50% ACN + 50% DI water) made it possible to directly apply eluted peptide solution on MALDI MS target plate [22–28].

### Mass spectrometry

Firstly, 1 μL chip-processed sample was spotted on the MALDI target plate and air-dried. Then 1 μL matrix (α-cyano-4-hydroxycinnamic acid (CHCA), 5 g/L) in 50% acetonitrile containing 0.1% TFA was spotted on the dried sample spot and allowed to co-crystallize. Mass spectra were acquired using a SHIMADZU AXIMA Resonance MALDI-IT-TOF equipped with a nitrogen laser emitting light at 337 nm, with an adjustable mass range of 800–4000 Da. The positive ion was detected under reflective mode. The spectrums of 500 laser shots were typically averaged to produce the final sample spectrum. The optimized accelerating voltage used was 50 kV. Data acquisition was controlled automatically by Shimadzu Biotech Launchpad program to deplete artifact bias.

### Rat serum handling procedure

Blood was collected from the eyes: elaborate of grown-up rats cultivated normally. All blood samples were maintained at room temperature (~25 °C) for 1 h to allow sample coagulation and then centrifuged at 4 °C for 15 min at 1400×*g*. The serum layer was aspirated and collected in polypropylene tubes to avoid the fluid in the buffy-coat layer. Samples were then aliquoted and stored at different storage conditions, except for the samples immediately analyzed.

#### Short-term storage

Fresh serum aliquots were stored either in an ice bath (0–4 °C) or at room temperature (25 °C). Samples stored first for 4, 8, 12, 24 h were analyzed at the indicated times.

#### Long-term storage

Fresh serum aliquots were stored either at room temperature (25 °C), at 4 °C, at −20 °C, at −80 °C, or in liquid nitrogen (−190 °C). At the appropriate times, aliquots were thawed at 4 °C, and subsequently prepared for analysis.

#### Freeze/thaw cycles

Four different freeze/thaw conditions were tested: −190 °C/25 °C, −190 °C/4 °C, −80 °C/25 °C, or −80 °C/4 °C. The serum samples were frozen down and kept at the indicated temperatures for 2 h, and then thawed at the indicated temperatures for 1 h and then immediately refrozen. This procedure was repeated for 1–5 times, amounting to 20 different freeze/thaw cycle conditions.

### Data processing and multivariate statistical analysis

Mass spectrometry data were converted into ASCII format on the integrated software of the SHIMDZU MALDI-TOF instrument (Shimadzu Biotech Launchpad). PCA analysis and significant difference analysis were performed on the commercially available program MarkerView version 1.2.1 version (AB SCIEX). PCA analysis was conducted using a non-supervised model, and for the scaling procedure we used a Pareto method.

## Results and discussion

### Performance of mesoporous chip as enrichment platform of LMW peptides in serum

**Fig. 1:**
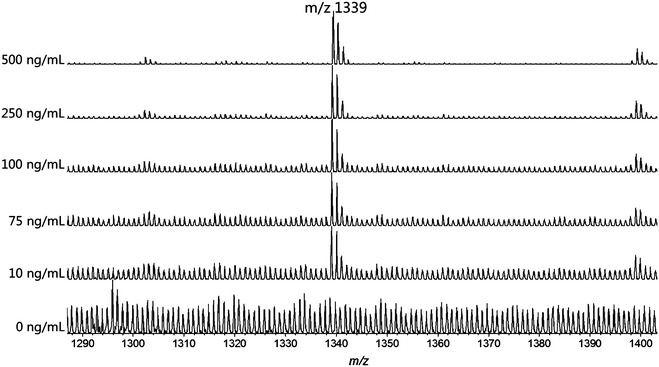
MALDI MS spectra of serums spiked with standard peptide (m/z 1339) at different concentrations (0, 10, 75, 100, 250, 500 ng/mL)

**Fig. 2:**
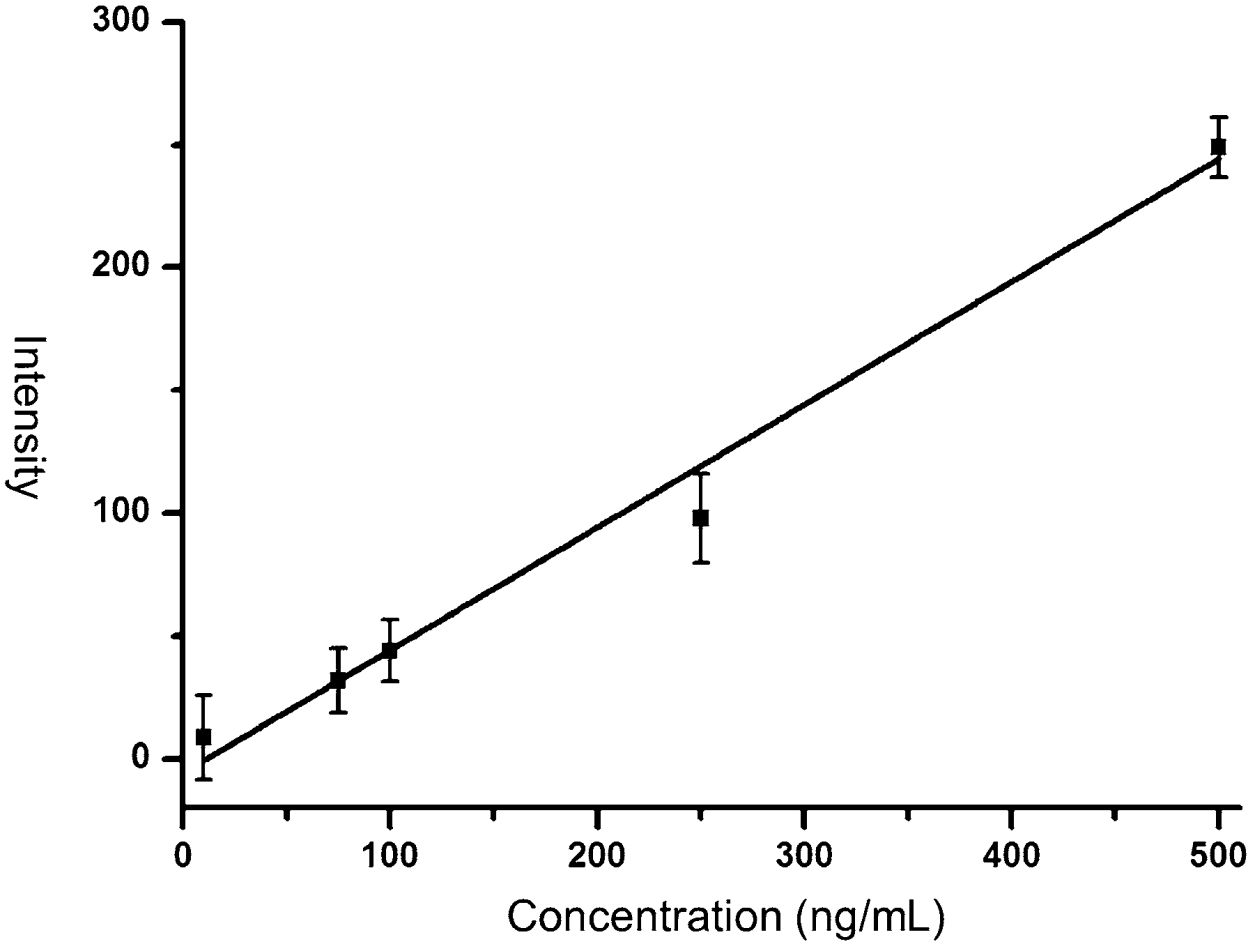
Linear correlation of MS intensities with peptide concentrations in the range from 10 to 500 ng/mL

In order to evaluate the sensitivity of the MS approach based on mesoporous chip enrichment, we tested serum samples spiked with synthesized standard peptide (Mw 1338) in different concentrations. As illustrated in Fig. 1, a strong signal for the standard peptide at m/z 1339 was detected at the concentrations tested. This standard peak was resolved from the background even at a concentration as low as 10 ng/mL. A good linear correlation with R^2^ = 0.996 was obtained in the range of 10–500 ng/mL, as shown in Fig. 2. These results confirmed the good performances of mesoporous chip-enabled MALDI MS approach in the relative quantitative analysis. Repeated test of one same serum sample in the different well on the mesoporous chip also showed good reproducibility, and the CV% of five endogenous m/z peaks from the peptide MS pattern respectively are 12.6% (m/z 1561.41), 6.6% (m/z 1553.37), 10.6% (m/z 1552.39), 5.8% (m/z 1394.66), 9.0% (m/z 897.62).

The performance of mesoporous silica chip to eliminate abundant large proteins has been detailed studied [22–28]. These works demonstrated that diverse LMW peptides (PI 4.0–10.2, molecular weight 800–4000 Da) could be stably harvested on the mesoporous silica chip [22, 28], and the abundant large proteins were efficiently eliminated [22–28]. Therefore, it is possible to apply this platform to sensitively acquire LMW peptide MS-pattern of bio-samples, and further estimate the variation of the different samples according to the MS pattern.

In our experiments, the whole sample preparation procedure was finished in 40 min in a high-throughput manner, with a very simple manipulation without repeated centrifugation, multiple fluid transfer, or column purification. This procedure significantly reduced the sample and labor consumption and increased the analysis throughput, therefore, should be suitable for the analysis of large number of samples, which should enable this method universally applicable in frontline clinical organizations such as hospital and bio-bank.

#### Impact of long-term storage condition on sample quality

It has been proven that the storage temperature exerts remarkable effects on the composition of bio-specimen stored for long-term [1, 19]. To verify if the peptide profiling enabled by meso-porous chip could express the effect caused by the stored temperature, we selected the fresh serum that just delivered from the living body as the standard specimen, and compared the MS patterns of samples stored under different environments. Five common storage environments were compared, including refrigerator cold closet (4 °C), refrigerator freezing chamber (−20 °C), ultra-low temperature freezer (−80 °C), liquid nitrogen (−190 °C) and at room temperature (25 °C).

**Fig. 3:**
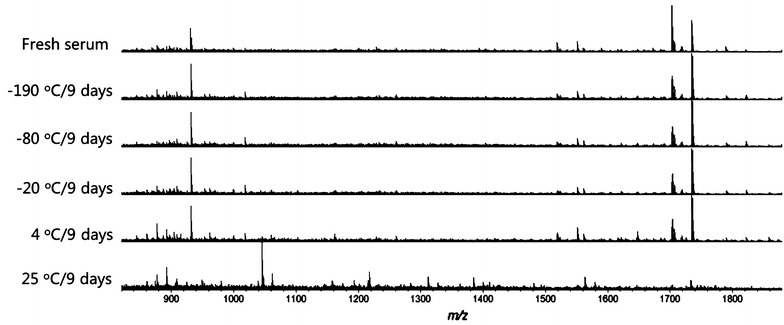
MALDI MS spectra of serums stored at different temperatures (−190, −80, −20, 4 and 25 °C) for 9 days and fresh collected serum

**Fig. 4:**
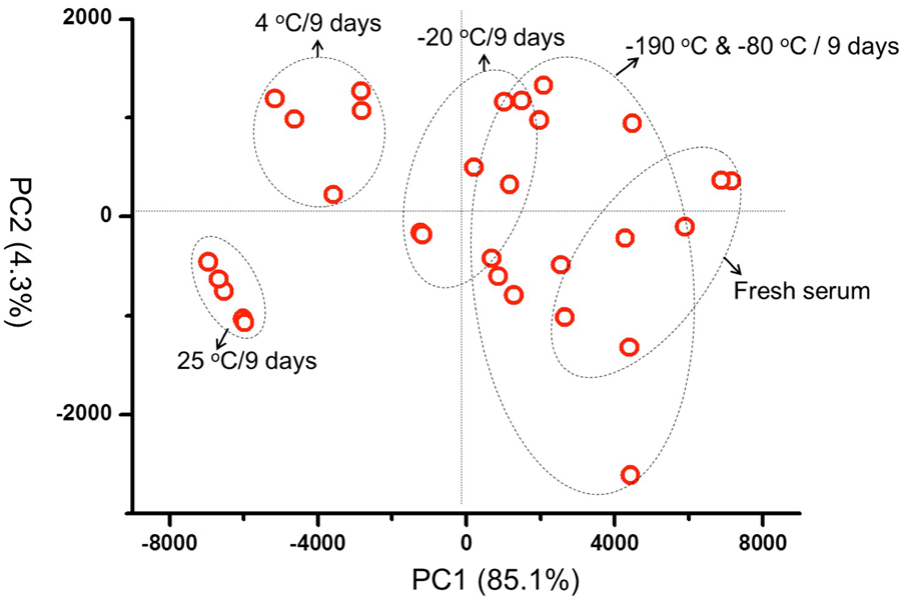
Principle composition analysis (PCA) of the different temperatures (−190, −80, −20, 4 and 25 °C) stored serums for 9 days

After 9 day storage, obvious changes were observed directly in the MS spectra of the serums at 25 °C (Fig. 3). A more distinct differentiation was illustrated in PCA analysis (Fig. 4). The 25 °C samples was the most distinctive group, far away from the samples stored at other temperatures. Meanwhile, the 4 °C group also showed a complete separation from the fresh samples, indicating that the 4 °C is also a destructive condition for the long-term storage. Comparatively, the MS patterns from −20, −80, and −190 °C groups are much closer or even overlapped to the fresh serum group, suggesting the key effect of freezing for the sample long-term storage. Lower temperature showed better conservation effects, as can be seen in −80 and −190 °C group, which just partially separated from the fresh group.

**Fig. 5:**
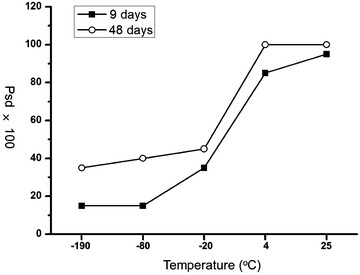
The percentage of peaks experienced significant difference at different temperatures (−190, −80, −20, 4 and 25 °C) for 9 days (*black square*) and 48 days (*white circle*)

To further investigate the extent of variation of samples at different conditions, we calculated the number of m/z peaks that indicated significant difference between stored samples and fresh samples (judgment standard: p value <0.01). As the temperatures rise, the percentage of peaks exhibiting significant difference (named as Psd value) also increased dramatically (Fig. 5): the Psd values of −190, −80, −20, 4 and 25 °C groups were 15, 15, 35, 85, and 95% respectively, which was coincide with the results of PCA analysis.

**Fig. 6:**
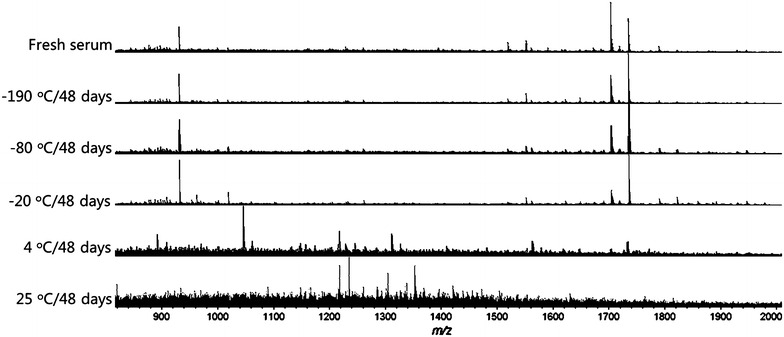
MALDI MS spectra of serums stored at different temperatures (−190, −80, −20, 4 and 25 °C) for 48 days and fresh collected serum

**Fig. 7:**
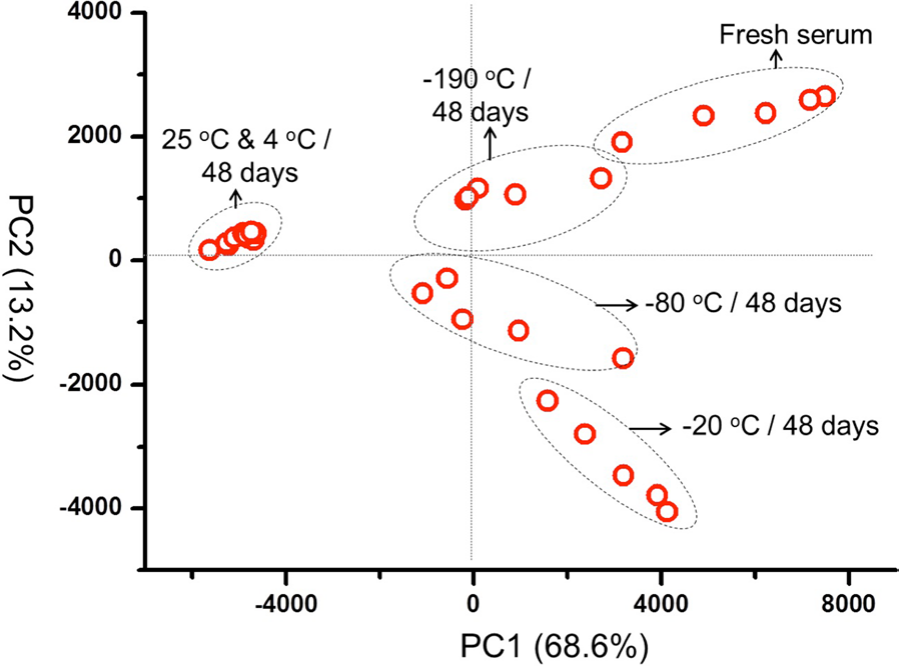
PCA of the serums stored at different temperatures (−190, −80, −20, 4 and 25 °C) for 48 days

This trend was further demonstrated from the analysis of samples stored for 48 days. The obvious changes of spectra can even be distinguished directly at the temperature of 4 and −20 °C (Fig. 6). In the PCA analysis, all the sample groups separated completely from the fresh samples except for the −190 °C group (Fig. 7), and the general freezing condition such as −20 °C is no longer appropriate. Besides, after stored for 48 day, the percentage of m/z peaks showing significant difference enhanced obviously compared with the samples stored 9 day, and still showed the highly dependent on temperatures (Fig. 5). To our surprise, the sample stored in ultra-low temperature freezer (−80 °C) also showed significant difference from the fresh samples and the Psd value reached 40%, which probably should be attributed to unstable temperature due to the frequent opening of the freezer door during this long period. These results suggested that as the storage time prolonged, a temperature-lowered and more stable condition is required.

It has been widely discussed that the proteases remained in bio-samples remarkably varied the proteome profile, especially at the enzyme-activated temperature [5, 29]. Besides, other influence factors such as the bacterial contaminate also emerge at relatively high temperature as time passes [29]. Thus, the storage temperature crucially influences the quality of long-term storage samples. We have discovered that the LMW peptide profiles enabled by the meso-porous chip were able to effectively represent the differentiation of samples stored at different temperatures, and the extent of variation of peptide spectra highly correlated with temperature, implying that this proposed approach can be utilized to evaluate the variation of sample quality caused by the handling procedure. The samples frozen at below −80 °C were customarily considered as stable and conserved samples. Based on the above analysis, it is concluded that in this chip-enabled measurement, the serum with a Psd value lower than 40% can be seen as a well preserved sample. From our experiments, liquid nitrogen should be the most ideal storage environment for the long-term storage of serum samples, and ultra-low temperature freezer could also be employed under the condition of temperature stabilized.

#### Impact of short-term storage conditions on sample quality

**Fig. 8:**
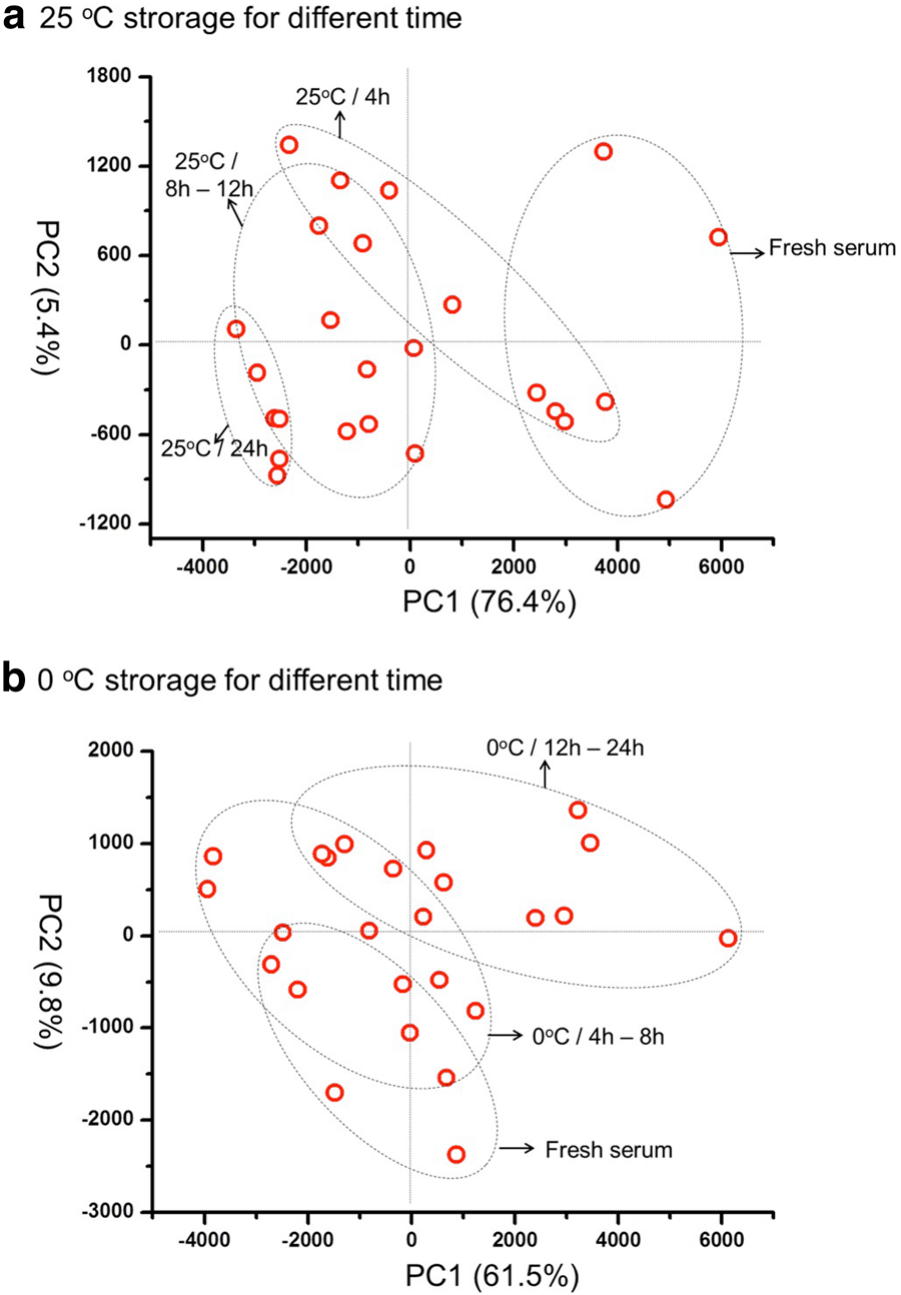
PCA of the serums stored under 25 °C (**a**) and 0 °C (**b**) for different times (0, 4, 8, 12, and 24 h)

**Fig. 9:**
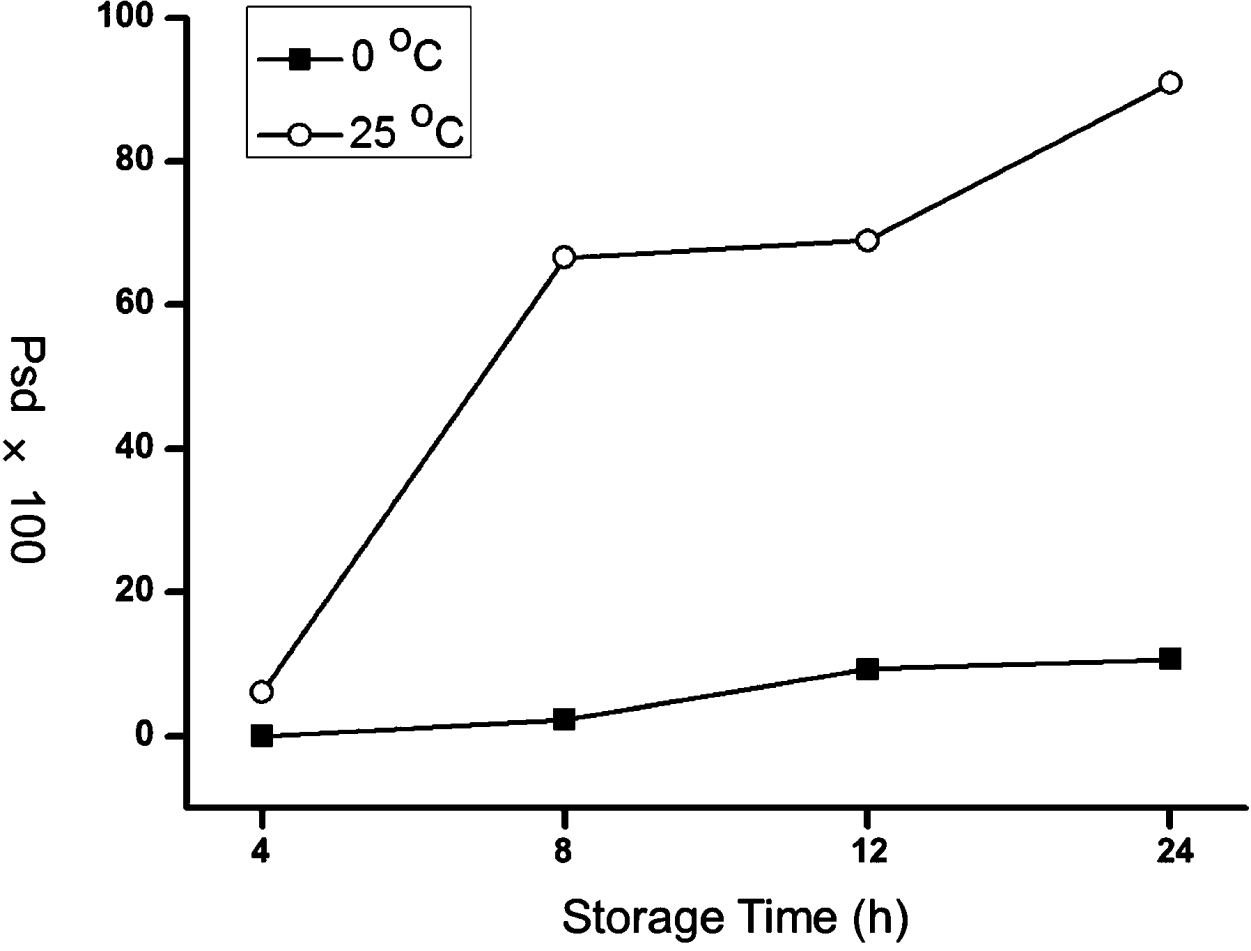
The percentage of peaks experienced significant difference in serums stored for different times (4, 8, 12, and 24 h) at 25 °C (*white circle*) and 0 °C (*black square*)

Clinical specimen just collected may not be able to be conserved in liquid nitrogen or freezer at the first time due to the lack of related instruments at the clinical frontlines, and it is probably inevitable to store samples at room temperature (25 °C) or ice bath (~0 °C) for a short while (<24 h). In order to optimize the short-term storage conditions, we analyzed the LMW peptide pattern of serums stored under these two temperatures for different times. As can be seen in PCA analysis (Fig. 8), at the both two temperatures, the alteration of peptide profiles enhanced as the storage time increase; meanwhile, the differentiation of 25 °C group (Fig. 8a) from fresh serum is much more evident than the 0 °C group (Fig. 8b). This is a theoretically reasonable result: the ice bath is a preferable storage condition compared with the room temperature due to the inactive enzyme and depressed hydrolyzing effect at lower temperature. A more clear results were showed by comparing the percentage of peaks indicating significant difference (Psd value), which increased gradually as the time passed under the both two temperature (Fig. 9). The Psd value was still lower than 11% (10.7%) even stored for 24 h at 0 °C, yet it was already over 60% when stored for 8 h at 25 °C. These results suggested that the best way to store serum for a short time is around 0 °C, and if the condition is unavailable, the time stored at room temperature should not be longer than 8 h.

#### Impact of freeze/thaw cycles on sample quality

**Fig. 10:**
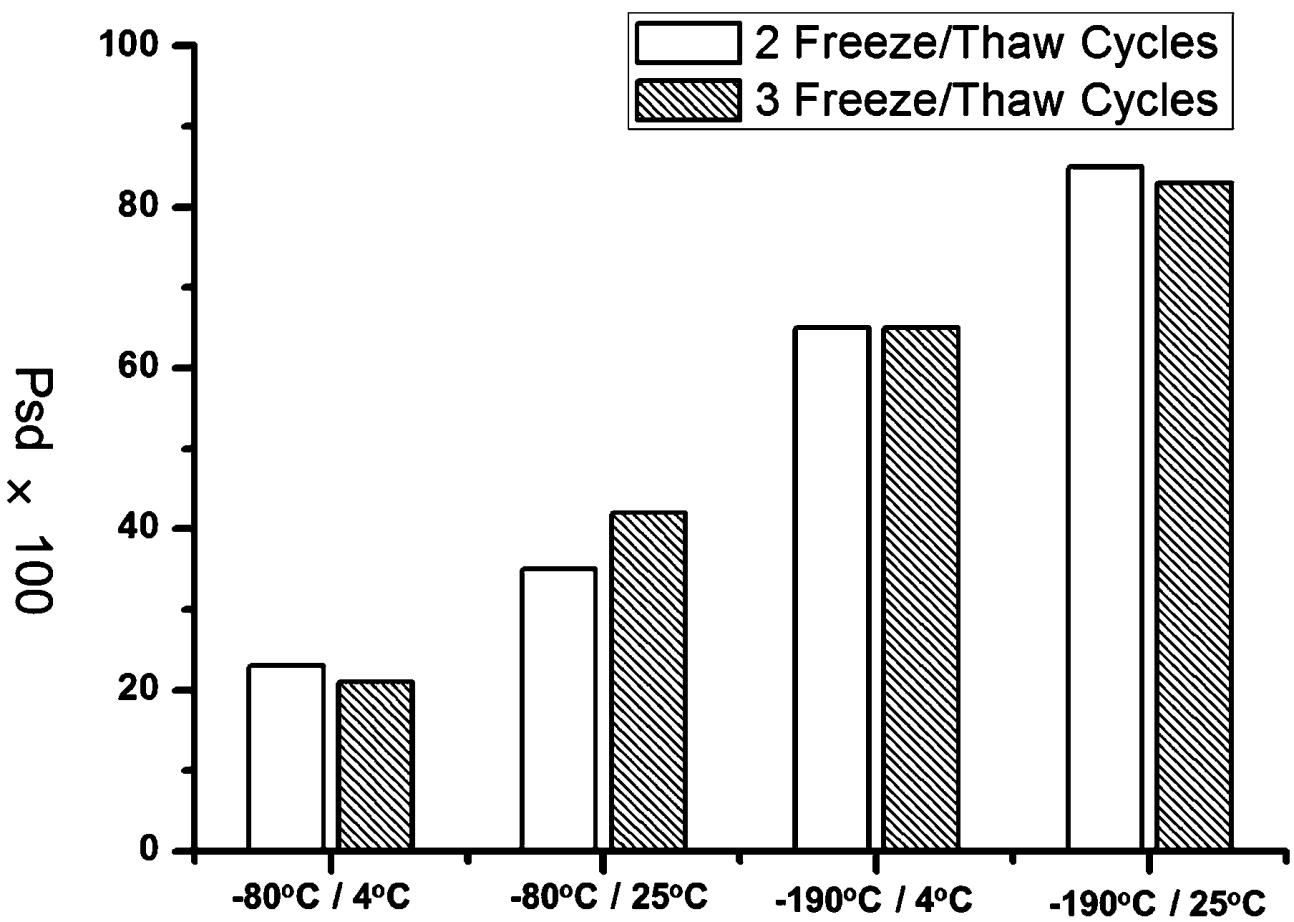
The percentage of peaks exhibiting significant difference in serums frozen/thawed at different temperatures for 2 and 3 cycles

Another important pre-analysis factor influencing the quality of samples is freeze/thaw handling, which is generally inevitable during from sample collecting to usage. It had been advised that freeze/thaw cycle altered the proteomics of serum [1]. To reduce this alteration, mesoporous chip-MS approach was used to investigate the serum cohort froze/thawed at different temperature conditions, including two common freezing-temperatures (−190 and 80 °C) and two thawing-temperatures (4 and 25 °C). As shown in Fig. 10, after 2 freeze/thaw cycles, the serums frozen at −80 °C exhibited lower Psd values compared with the condition of −190 °C. Meanwhile, the serums thawed at 4 °C showed lower Psd values than 25 °C. Among the four freeze/thaw conditions tested here (Fig. 10), the best conservation of serums with lowest Psd value was the condition of freezing at −80 °C/thaw at 4 °C. Similar results were observed after 3 cycles of freeze/thaw handling (Fig. 10).

It had been reported that freeze/thaw handling altered the composition of serum mainly due to the protein/peptide aggregation, precipitation and adsorption to tube-wall during this procedure [1]. Thus, it can be deduced that the over-fast freezing at −190 °C highly increases the aggregation and precipitation of proteins (peptide), changing more composition of serum than −80 °C. On the other hand, fast thawing at 25 °C activates the enzymes in serum, resulting in more hydrolysis to the serum proteome than 4 °C. Thus, slow freezing at −80 °C and thaw at 4 °C should be selected during the storage and test of serum, to obtain a best conservation to the protein/peptides. Considering the liquid nitrogen is still the optimized environment for the long-term storage as we proved above, it is recommended that the bio-fluid could be frozen firstly at −80 °C, and then transferred to liquid nitrogen for the long-term storage.

**Fig. 11:**
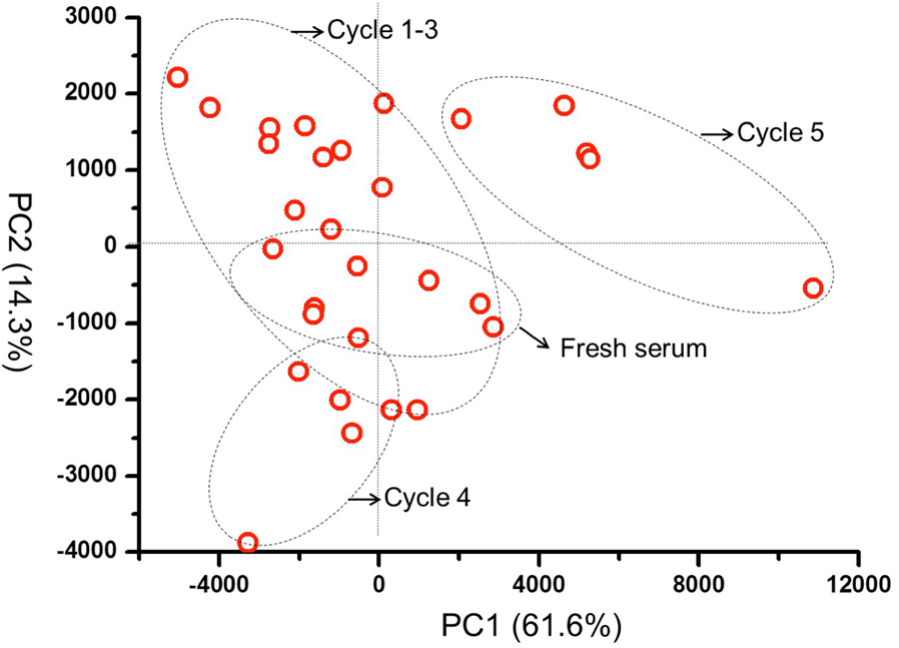
PCA of serums froze at −80 °C and then thawed at 4 °C for 1–5 cycles

**Fig. 12:**
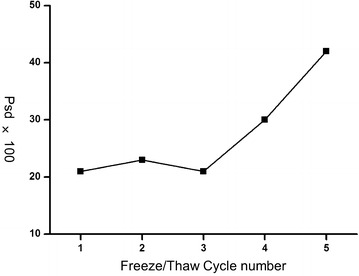
The percentage of peaks exhibiting significant difference in serums frozen at −80 °C and then thawed at 4 °C for 1–5 cycles

Afterwards, we further investigated the impact of freezing frequency. Serums were repeatedly froze at −80 °C and then thawed at 4 °C for 1–5 cycles, and then compared with the original fresh serums. As shown in PCA analysis (Fig. 11), when the cycles of freezing/thaw were lower than three times, the frozen/thawed serums were hard to discern from fresh serums. When the freezing/thaw cycles exceeded 4 times, the frozen/thawed serums were separated from the fresh serums completely (Fig. 11). The same trends can also be concluded from the comparison of Psd values (Fig. 12). The Psd values were almost constant and only around 20% when freezing times were less than 3. However, as freezing times increased to 5, the Psd value was over 40%, implying a serious change of serum proteome (Fig. 12).

Proteins in serum tended to aggregate and participate during freezing procedure [1]. These aggregations of proteins were getting hard to re-dissolve after serums were repeatedly frozen and thawed, resulting in the change of serum proteome. Therefore, to preserve the original constituents in serum, the freezing/thaw handling to serum should be as few as possible. As we studied, for the serums used for proteomics research, freezing/thaw cycles shouldn’t be over three times.

## Conclusions

In summary, through the mesoporous material assisted MALDI MS approach, we have successfully measured the alteration of LMW peptides in serums caused by different handling condition, and identified the sample groups that is handled under inappropriate conditions and no longer suitable for further clinical experiments. This indicates that the method presented here does offer the critical information on sample composition that is highly related to the handling procedure. On the basis of these findings, we propose that this method can be used as an effective evaluation tool for the controlling of sample quality. More importantly, this simple and fast method should be particularly useful to a wide application in clinical settings, where quality tests for a large amount of samples before clinical practices are in great demand. Besides the serum samples, this approach should be extendable to the evaluation of other fluid bio-samples containing peptides, such as urea, saliva or cerebrospinal fluid.

## Abbreviations

LMW: low molecular weight
MALDI MS: matrix assisted laser desorption ionization mass spectrometry
PCA: principle composition analysis
TFA: trifluoroacetic acid
ACN: acetonitrile
CHCA: α-cyano-4-hydroxycinnamic acid
ELISA: enzyme-linked immunosorbent assay
CE: capillary electrophoresis
PAGE: polyacrylamide gel-electrophoresis
Psd value: the percentage of MS peaks exhibiting significant difference
